# D-xylose suppresses hepatocellular carcinoma progression by regulating dihydrodiol dehydrogenase and remodeling the immune microenvironment

**DOI:** 10.3389/fimmu.2026.1792196

**Published:** 2026-03-13

**Authors:** Haiyang Yu, Xiangxiang Wu, Yiting Liu, Congling Xin, Xiaoxia Guo, Yu Zhou, Xiaoyi Ding

**Affiliations:** 1 Department of Radiology, Ruijin Hospital, Shanghai Jiao Tong University School of Medicine, Shanghai, China; 2 Department of Radiology, Lianyungang TCM Hospital Affiliated to Nanjing University of Chinese Medicine, Lianyungang, China; 3 Department of Gynecology, Fudan University Shanghai Cancer Center Minhang District, Shanghai, China

**Keywords:** DHDH, D-xylose, HCC, NADPH/ROS axis, time

## Abstract

**Introduction:**

Hepatocellular carcinoma (HCC) is a leading cause of cancer-related death with limited treatment options. Dihydrodiol dehydrogenase (DHDH), an enzyme involved in D-xylose metabolism, has unclear roles in tumorigenesis and immune regulation. This study aims to investigate the clinical significance, biological functions, and immunomodulatory mechanisms of DHDH in HCC, and to explore the therapeutic potential of targeting its metabolic activity.

**Methods:**

DHDH expression and its clinical correlation were analyzed using TCGA-LIHC data and validated in HCC tissue microarrays. *In vitro* functional assays were performed using DHDH-overexpressing and knockdown Hepa1–6 cells. Immune interactions were assessed via co-culture with CD8^+^ T cells and flow cytometry. Subcutaneous tumor models in immunodeficient and immunocompetent mice, alongside HCC organoid models, were used to evaluate tumor growth and immune microenvironment changes. The therapeutic effect of D-xylose, alone or combined with anti-PD-L1, was examined *in vivo*.

**Results:**

DHDH was highly expressed in HCC tissues and significantly associated with poor prognosis. Functional studies demonstrated that DHDH overexpression promoted HCC cell proliferation and invasion while suppressing CD8^+^ T cell activity, potentially through the upregulation of PD-L1 and downregulation of β2-microglobulin (B2M). Further investigations revealed that D-xylose, a metabolic substrate of DHDH, significantly enhanced intracellular NADPH production and reduced ROS levels, thereby alleviating oxidative stress-induced T cell dysfunction. In patient-derived organoid-PBMC co-culture models, D-xylose treatment markedly enhanced T cell immune activity.

**Discussion:**

DHDH drives HCC progression and immune evasion by promoting an immunosuppressive microenvironment. Targeting DHDH with D-xylose restores CD8^+^ T cell function and synergizes with immunotherapy.

## Graphical Abstract

**Figure:**
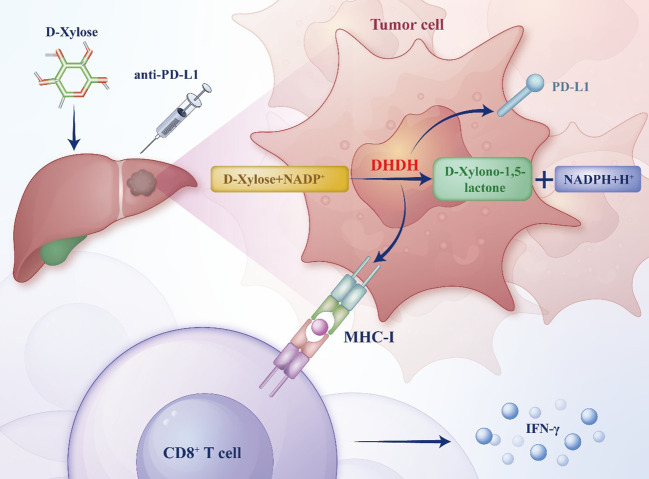

## 1 Introduction

Hepatocellular carcinoma (HCC) is the most common type of primary liver cancer, accounting for the vast majority of primary liver malignancies, and is characterized by marked heterogeneity and aggressiveness. According to the GLOBOCAN 2022 statistics released in 2024, liver cancer accounted for 4.3% of all newly diagnosed cancer cases and 7.8% of all cancer-related deaths worldwide in 2022, ranking third among all malignancies. Notably, approximately 72% of newly diagnosed cases and deaths occur in Asia, including China—owing to its large population, which is one of the heaviest global burdens of liver cancer (1, 2). In recent years, immune checkpoint inhibitors (ICIs) combined with anti-angiogenic therapies have emerged as first-line treatment options for advanced HCC, significantly improving survival outcomes in select patients (3). However, most patients experience either a primary nonresponse or acquired resistance (4). A major barrier to the efficacy of immunotherapy is the inherent immunotolerant tumor immune microenvironment (TIME) in HCC, and identifying and targeting the key mechanisms driving this immunosuppressive niche is critical for optimizing immunotherapeutic strategies (5–8).

Metabolic reprogramming is a hallmark of tumor progression and has been increasingly recognized as a key modulator of the TIME (9). By rewiring glucose, lipid, and amino acid metabolism, tumor cells not only fulfill their high demands for energy and biosynthetic precursors but also exert systemic immunomodulatory effects through metabolic competition, accumulation of immunosuppressive metabolites, and redox regulation (9–11). For instance, glucose competition impairs the acquisition of essential metabolic substrates by T cells, whereas lactate, an end product of aerobic glycolysis, can promote T cell expansion but simultaneously suppress cytotoxic T lymphocyte (CTL) and natural killer (NK) cell activity. Additionally, aberrant lipid metabolism generates cholesterol-derived ligands that activate the liver X receptor (LXR) pathway and downregulate major histocompatibility complex class I (MHC-I) expression, thereby impairing antigen presentation (12). Reactive oxygen species (ROS), another crucial intermediary linking metabolism and immunity, contribute to dendritic cell dysfunction and promote T cell exhaustion when excessively accumulated (13, 14). Collectively, these findings underscore the profound impact of tumor metabolic states on immune surveillance and elimination, positioning metabolic pathways as critical targets for enhancing anti-tumor immunity.

Within the metabolism-immunity interaction network, the NAD(P)^+^/NAD(P)H system serves as a central axis regulating cellular redox homeostasis and plays a pivotal role in maintaining T cell function (15). Dihydrodiol dehydrogenase (DHDH), a member of the NAD(P)^+^-dependent short-chain dehydrogenase/reductase family, catalyzes the oxidation of various alcohol substrates (16, 17). Emerging studies have shown that DHDH mediates the metabolism of pentose sugars such as D-xylose, a process coupled with the generation of reduced nicotinamide adenine dinucleotide phosphate (NADPH) (18). As a naturally fermentable sugar, D-xylose can be selectively taken up and enters the pentose phosphate pathway (PPP) *in vivo*, where it is metabolized by DHDH to produce NADPH (18). Notably, NADPH is not only a critical regulator of ROS clearance and cellular antioxidant defense but is also essential for preserving T cell redox balance, preventing exhaustion, and sustaining effector functions (19). Therefore, DHDH-mediated D-xylose metabolism may exert dual immunometabolic effects: on the one hand, modulating the redox state to influence tumor cell biology, and on the other, promoting metabolic fitness of immune effector cells to enhance antitumor responses. Although preliminary evidence in breast cancer suggests that DHDH may facilitate immune evasion, its expression pattern, immunomodulatory role, and impact on immunotherapeutic responsiveness in HCC remain largely unexplored (18).

Given this background, this study aimed to investigate the expression pattern and immunometabolic role of DHDH in HCC. We further evaluated whether D-xylose, as a substrate of DHDH, can reshape the TIME and enhance immunotherapy response. Using multi-level approaches spanning molecular, cellular, organoid, and *in vivo* models, we systematically elucidated the function of DHDH and explored its potential as a target for metabolic immunotherapy. Our findings provide a rationale for combining metabolic modulation with immune-based strategies in HCC.

## 2 Materials and methods

### 2.1 TCGA database analysis

RNA sequencing data and corresponding clinical information of patients with HCC were obtained from the TCGA-LIHC cohort (**Supplementary Table 1**). Data analyses were performed using the R software (v4.2.1) and the Xiantao Academic Toolkit. The Wilcoxon rank-sum test was used to assess the differential expression of DHDH between the tumor and normal tissues. Single-sample gene set enrichment analysis (ssGSEA) was used to quantify immune cell infiltration. The correlation between DHDH expression and various immune checkpoint molecules (PDCD1 and CD274) was evaluated. Kaplan-Meier survival curves, univariate and multivariate Cox regression analyses were conducted using the Xiantao Academic Toolkit and RStudio.

### 2.2 HCC tissue microarrays

Two sets of HCC tissue microarrays (TMAs) were used for immunohistochemical (IHC) staining of DHDH and Ki-67. Clinical data corresponding to the patients represented in the TMAs, including age, sex, vascular invasion status, and survival outcomes, were collected to assess the association between protein expression and clinicopathological features (**Supplementary Table 2**). This study was approved by the institutional ethics committee (Approval No. HLivH180Su30).

### 2.3 Cell lines and culture

Murine Hepa1–6 hepatocellular carcinoma cells were purchased from Suzhou Haixing Bioscience Co., Ltd. and cultured in high-glucose Dulbecco’s modified Eagle’s medium (DMEM, Corning, USA) supplemented with 10% fetal bovine serum and 1% penicillin-streptomycin (Beyotime, China) at 37 °C in a humidified incubator containing 5% CO_2_.

To generate stable cell lines, the PiggyBac transposon system was employed. For overexpression (oeDHDH), the full-length murine Dhdh cDNA (NCBI Reference Sequence: NM_027903.4) was cloned into the PB-CAG-MCS-EF1α-Puro vector. For knockdown (shDHDH), short hairpin RNA (shRNA) sequences targeting murine Dhdh (or a non-targeting scrambled control) were cloned into the PB-U6-MCS-EF1α-Puro vector. shDHDH target sequence: 5’-CGAGGAGTTCGCACAGAAATT-3’, shCtrl target sequence: 5’-CCTAAGGTTAAGTCGCCCTCG-3’. Hepa1–6 cells were seeded in 6-well plates at a density of 2×10^5^ cells per well and co-transfected with the respective transposon plasmid and the HyPBase transposase expression plasmid (at a mass ratio of 5:1) using Lipofectamine 3000 reagent according to the manufacturer’s protocol. Forty-eight hours post-transfection, the culture medium was replaced with fresh complete medium containing 8 μg/mL puromycin for selection. The selection medium was refreshed every 2–3 days until resistant foci emerged. DHDH expression levels were validated using quantitative PCR and Western blot analyses.

### 2.4 Quantitative real-time PCR

Quantitative real-time PCR (qRT-PCR) was performed to assess the expression levels of the DHDH gene. Total RNA was extracted using TRIzol reagent (Thermo Fisher Scientific, USA), and the RNA concentration was determined by measuring the optical density (OD) at 260 nm and 280 nm using a spectrophotometer. A total of 100 ng of RNA was reverse-transcribed into complementary DNA (cDNA), which was used as the template for qRT-PCR amplification. Primers were designed using the Primer 5 software. GAPDH served as the internal control, and the relative DHDH expression was calculated using the 2^^-ΔΔCt^ method. The primer sequences are listed in **Supplementary Table 3**.

### 2.5 Western blotting

Total protein was extracted from cells using RIPA lysis buffer (Beyotime, China), and protein concentrations were determined using the BCA assay. The protein lysate (100 μL) was mixed with 25 μL of 5× loading buffer, boiled for 10 min, and then cooled. Equal volumes of protein samples were separated by SDS-PAGE using 4-20% gradient gels at 120 V for 40 min, followed by transfer onto 0.45 μm polyvinylidene fluoride (PVDF) membranes (Millipore, USA). The membranes were blocked for 1 h with blocking buffer and incubated overnight at 4 °C with primary antibodies against: DHDH (1:8000, 14877-1-AP, Protintech), β2-microglobulin (B2M) (1:5000, ab75853, Abcam), PD-L1(1:1000, ab205921, Abcam), and Vinculin (1:10000, ab129002, Abcam). HRP-conjugated goat anti-rabbit/mouse IgG (Cell Signaling Technology, Danvers, MA, USA) was used as the secondary antibody. Protein bands were visualized using ECL chemiluminescence reagent (Advansta, USA, Cat#K-12045-D20) and quantified using ImageJ software. Data were normalized to the corresponding control group (oeCtrl or shCtrl, set to 1) in the overexpression and knockdown systems, respectively.

### 2.6 Cell proliferation assay

Cell viability was assessed using a CCK-8 kit (Beyotime, Shanghai, China). Briefly, cells were seeded in 96-well plates and incubated for 0, 24, 48, and 72 h. At each time point, 10 μL of CCK-8 solution was added to each well along with 90 μL of complete medium. After 2 h of incubation at 37 °C in a humidified atmosphere containing 5% CO_2_, optical density (OD) was measured at 450 nm using a microplate reader (BioTek Synergy H1, Winooski, VT, USA).

### 2.7 Wound healing assay

Cells were seeded into 6-well plates at a density of 5 × 10^5^cells per well. Horizontal reference lines were drawn on the underside of the plate by using a marker. After the cells had adhered and reached confluence, a straight scratch was made perpendicular to the horizontal lines using a sterile pipette tip. The wells were washed three times with PBS to remove detached cells, and fresh DMEM (Corning, USA) was added. The cells were incubated at 37 °C in a 5% CO_2_ incubator, and wound closure was imaged at 0, 24, and 48 h.

### 2.8 Transwell invasion assay

Matrigel matrix (Corning, USA) was diluted with serum-free medium at a ratio of 1:8 and used to pre-coat the upper chambers of Transwell inserts (8 μm pore size). The coated chambers were incubated at 37 °C for 3 h to allow gel solidification. Cells were resuspended in serum-free medium at a density of 2 × 10^5^ cells/mL, and 100 μL of the suspension was added to the upper chambers. After 24 h of incubation at 37 °C and 5% CO_2_, the inserts were fixed with 4% paraformaldehyde for 30 minutes and stained with 0.1% crystal violet (​​Beyotime, China) for 5 minutes. The invasive cells on the lower surface of the membrane were visualized and photographed under a microscope. Each assay was performed in triplicates.

### 2.9 CD8^+^ T cell co-culture assay

​​CD8^+^ T cells were isolated from splenocytes of C57BL/6 mice using a mouse CD8^+^ T Cell Isolation Kit (Thermo Fisher Scientific, USA) according to the manufacturer’s protocol. Isolated CD8^+^ T cells were activated with Dynabeads™ Mouse T-Activator CD3/CD28 (Thermo Fisher Scientific, USA) at a bead-to-cell ratio of 1:1 for 24 hours prior to co-culture. For the co-culture assay, pre-activated CD8^+^ T cells were harvested and co-cultured with target tumor cells (Hepa1-6) at an effector-to-target (E:T) ratio of 5:1 in 24-well plates. The co-culture system was maintained in DMEM medium supplemented with 10% FBS and 1% penicillin-streptomycin at 37 °C in a humidified incubator with 5% CO_2_ for 48 h. Following incubation, the supernatant was collected by centrifugation at 300 × g for 5 minutes. The concentration of interferon-gamma (IFN-γ) in the supernatant was quantified using a mouse IFN-γ ELISA kit (Beyotime, China) according to the manufacturer’s instructions. The absorbance was measured at 450 nm with a wavelength correction set to 570 nm using a microplate reader (BioTek Synergy H1, Winooski, VT, USA).

### 2.10 Flow cytometry

For flow cytometry analysis, single-cell suspensions were first washed with PBS and resuspended in staining buffer. Fluorochrome-conjugated antibodies (CD45, CD8, IFN-γ, H-2Kb, and B2M) were added and incubated at 4 °C in the dark for 30 min. After staining, the cells were washed, resuspended, and analyzed using a flow cytometer. Data were processed using FlowJo software (v10.8.1, TreeStar). The gating strategy is detailed in **Supplementary Materials** (**Supplementary Figure 2F**).

### 2.11 *In vivo* mouse studies

All animal experiments were conducted in accordance with the institutional guidelines for the care and use of laboratory animals and were approved by the Institutional Animal Ethics Committee (Approval No. WTPZ20250207001). Six- to eight-week-old C57BL/6 mice and immunodeficient nude mice were purchased from Shanghai Wuchuang Biotechnology Co., Ltd, and housed under specific pathogen-free (SPF) conditions. All procedures were performed to minimize animal suffering and in compliance with the “3Rs” principle (Replacement, Reduction, Refinement).

A total of 0.1 mL of cell suspension (2 × 10^7^ cells/mL) from oeDHDH, DHDH wild-type (DHDH-WT), or shDHDH cells was subcutaneously injected into C57BL/6 mice or immunodeficient nude mice. Tumor volumes were measured every three days after tumor establishment. Mice were euthanized by cervical dislocation when the tumor volume approached 2,000 mm³, and tumor tissues were harvested for further analysis.

For monotherapy evaluation in the oeDHDH-derived xenograft model, mice were randomized into two groups: (1) control group (oral gavage of saline, 50 mg/kg/day) and (2) D-xylose group (oral gavage of D-xylose, 50 mg/kg/day). For combination therapy studies, mice were randomized into four groups:

1. control (saline, 50 mg/kg/day, oral gavage),
2. D-xylose monotherapy (50 mg/kg/day, oral gavage),
3. anti-PD-L1 monotherapy (10 mg/kg, intraperitoneal injection every 3 days),

and (4) D-xylose plus anti-PD-L1 (D-xylose, 50 mg/kg/day, oral gavage; anti-PD-L1, 10 mg/kg every three days, intraperitoneally).

### 2.12 Hematoxylin and eosin staining and immunohistochemistry

Tumor tissues were fixed in 4% paraformaldehyde for 24 h, embedded in paraffin, and sectioned using standard protocols. For HE staining, sections were deparaffinized, rehydrated, sequentially stained with hematoxylin, differentiated in acid alcohol, blued, counterstained with eosin, dehydrated, and mounted. The histological morphology was examined and imaged under a microscope.

For IHC, endogenous peroxidase activity was blocked using 3% hydrogen peroxide (H_2_O_2_), followed by blocking with serum. The sections were then incubated overnight at 4 °C with primary antibodies. The next day, horseradish peroxidase-conjugated secondary antibodies were applied, followed by DAB chromogenic development, hematoxylin counterstaining, dehydration, and mounting. Whole-slide images were obtained using a digital scanner. Quantitative analysis of the entire tissue section was performed using the Aipathwell pathological image analysis software (Servicebio, Wuhan, China).

### 2.13 HCC organoids

HCC organoids were established using a three-dimensional Matrigel-based culture system. Stable DHDH-overexpressing organoid lines were generated via lentiviral transduction with empty vector-transduced organoids serving as controls. The efficiency of DHDH overexpression was validated by quantitative real-time PCR (qRT-PCR). Organoid viability was assessed using ATP-based luminescence assay.

For immune co-culture experiments, human peripheral blood mononuclear cells (PBMCs) were isolated and T cells were expanded through CD3/CD28 stimulation. These T cells were then co-cultured with DHDH-overexpressing organoids at an E:T ratio of 5:1. The treatment groups included a saline control, D-xylose alone, anti-PD-L1 antibody alone, and a combination of D-xylose with anti-PD-L1. After 72 h of co-culture, organoid viability was evaluated using the ATP assay.

Flow cytometry was performed to analyze the changes in the immune microenvironment. Cells harvested from the co-culture system were subjected to viability staining, followed by surface and intracellular staining for CD45, CD8, IFN-γ, PD-L1, and B2M. Data were analyzed using the FlowJo software. This study was approved by the Institutional Ethics Committee (Approval No. CTOB-2025-0709).

### 2.14 Measurement of NADP^+^/NADPH levels

To accurately assess intracellular NADPH and NADP^+^ levels, oeDHDH cells were co-cultured with CD8^+^ T cells and divided into control and treatment groups supplemented with 200 μM D-xylose. After 24 h of incubation, oeDHDH cells were harvested and sample preparation and colorimetric analysis were performed according to the manufacturer’s protocol using the NADP^+^/NADPH Quantification Kit (WST-8 method, MCE, HY-K1501). To specifically determine NADPH levels, a portion of each sample was heated at 60 °C for 30 min to decompose NADP^+^. Non-heated samples were used to quantify the total NADP(H) levels. The NADP^+^ concentration was calculated by subtracting NADPH from total NADP(H), and the NADP^+^/NADPH ratio was subsequently derived.

### 2.15 Measurement of ROS

The co-culture system described in Section 2.14 was used. oeDHDH cells were co-cultured with CD8^+^ T cells in the presence or absence of 200 μM D-xylose for 24 h, after which oeDHDH cells were collected. Intracellular ROS levels were measured using the fluorescent probe DCFH-DA according to the protocol of the ROS Detection Kit (MCE, HY-K0320). To further evaluate the immunological consequences of ROS changes, IFN-γ levels were simultaneously quantified in the co-culture supernatants.

### 2.16 Evaluation of ROS-mediated suppression of T cell function

To investigate the effect of ROS on T cell function, a co-culture system comprising oeDHDH cells and CD8^+^ T cells was established. During T cell activation, 50 or 100 μM hydrogen peroxide (H_2_O_2_) was added to artificially elevate ROS levels in tumor cells; the control group received no H_2_O_2_ treatment. After 48 h of co-culture, supernatants were collected and analyzed for IFN-γ secretion using an ELISA kit. In parallel, tumor cells were harvested to assess ROS levels, confirming the efficacy of H_2_O_2_ treatment.

### 2.17 Statistical analysis

Statistical analyses were performed using GraphPad Prism 9.0 and R software (v4.2.2). A two-sided P value < 0.05 was considered statistically significant. Significance levels are denoted as follows: *P* ≥ 0.05, not significant (ns); * *P* < 0.05; ** *P* < 0.01; *** *P* < 0.001.

Data distribution was assessed using the Shapiro–Wilk test for normality and Levene’s test for homogeneity of variance. Continuous variables are presented as mean ± standard error of the mean (SEM) when normally distributed and as median with interquartile range (IQR) when normality assumptions were not met.

For comparisons between two groups, an unpaired Student’s t test was used when parametric assumptions were satisfied; otherwise, the Mann–Whitney U test was applied. For comparisons involving more than two groups, one-way analysis of variance (ANOVA) followed by Tukey’s *post hoc* multiple comparisons test was performed for normally distributed data with equal variances, whereas the Kruskal–Wallis test was used for non-parametric data. For analyses involving multiple simultaneous comparisons (e.g., evaluation of multiple immune cell subsets or immune checkpoint molecules), P values were adjusted using the Benjamini–Hochberg procedure to control the false discovery rate (FDR).

Survival outcomes were analyzed using Kaplan–Meier curves and compared using the log-rank test. Prognostic factors were initially evaluated using univariate Cox proportional hazards regression analysis, and variables with a P value < 0.10 were subsequently entered into the multivariate Cox model to identify independent prognostic factors.

Clinical variables with incomplete annotation were handled using an available-case (complete-case per analysis) approach. For each statistical analysis, all subjects with non-missing values for the variables of interest were included. No additional exclusions were applied beyond missing data inherent to the original datasets, and multiple imputation was not performed. The effective sample size for each analysis is reported in the corresponding tables.

Associations between gene expression and clinical parameters were assessed using the chi-square (χ²) test or Spearman correlation analysis. Unless otherwise specified, all *in vitro* cellular experiments were independently repeated at least three times.

## 3 Results

### 3.1 DHDH is upregulated in HCC and correlates with immune cell infiltration and immune checkpoint molecules

**Figure 1:**
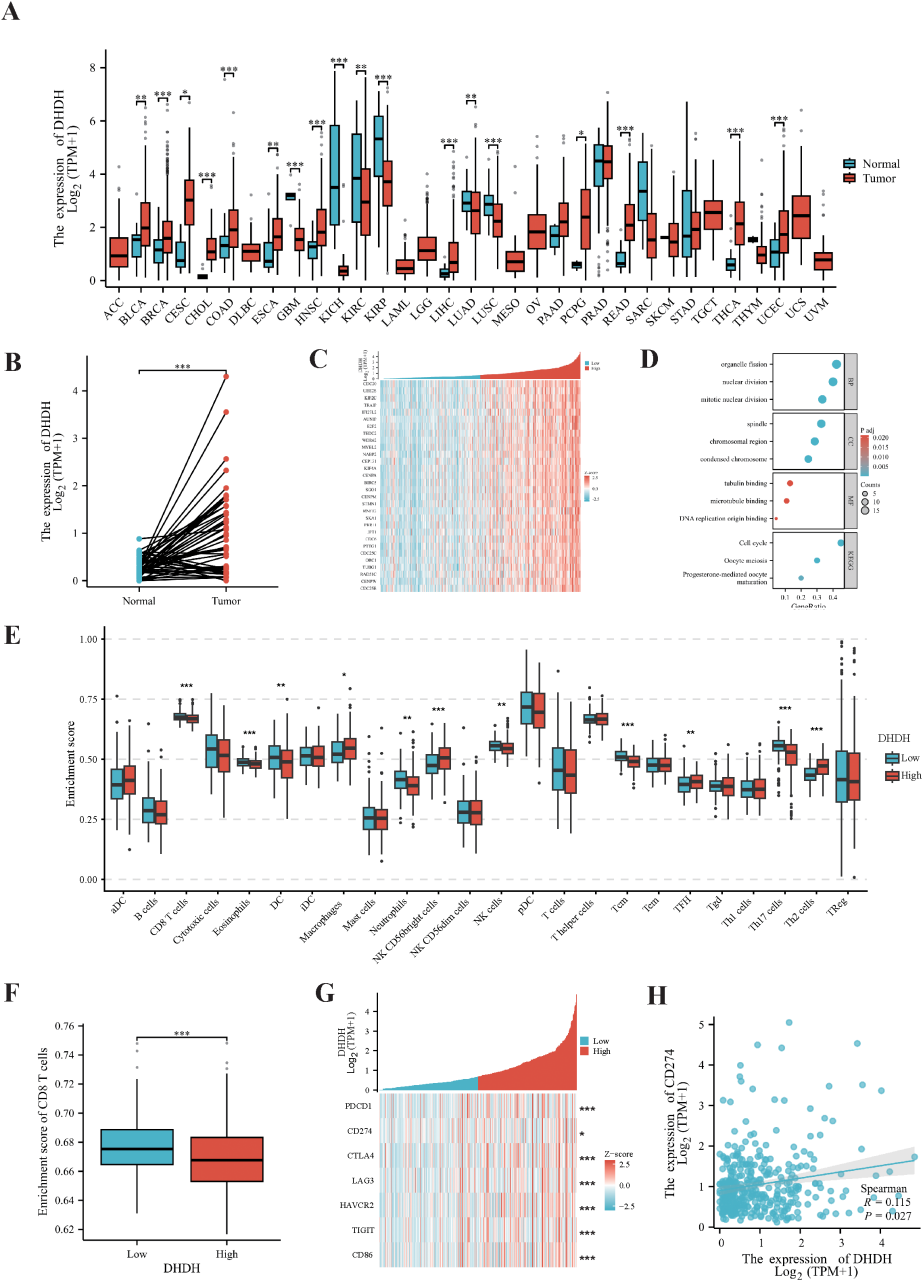
DHDH is upregulated in HCC and associated with an immunosuppressive microenvironment. **(A, B)** Pan-cancer and LIHC cohort analyses from TCGA reveal that DHDH expression is significantly elevated in liver cancer and multiple tumor types compared with normal tissues. **(C)** Hierarchical clustering based on DHDH expression shows distinct transcriptomic profiles between high and low expression groups. **(D)** GO enrichment analysis of DHDH co-expressed genes indicates involvement in cell cycle-related pathways. **(E, F)** ssGSEA analysis shows reduced infiltration of CD8^+^ T cells, activated dendritic cells (aDCs), and NK cells, but increased Tregs in the high DHDH group. **(G)** Immune checkpoint molecules including PDCD1, CD274, CTLA4, and LAG3 are significantly upregulated in tumors with high DHDH expression. **(H)** DHDH expression positively correlates with CD274 levels. * represents P < 0.05; ** represents P < 0.01; *** represents P < 0.001.

To systematically evaluate the expression pattern of DHDH across various tumor types, we analyzed RNA sequencing data from The Cancer Genome Atlas (TCGA) pan-cancer cohorts. DHDH expression was significantly upregulated in multiple malignancies, including liver hepatocellular carcinoma (LIHC), cholangiocarcinoma (CHOL), and pancreatic adenocarcinoma (PAAD), compared with the corresponding normal tissues (**Figure 1A**). ​Consistently, in paired samples from the LIHC cohort, DHDH expression was markedly higher in tumor tissues than in the adjacent normal tissues (**Figure 1B**). Transcriptomic profiling of LIHC samples revealed distinct expression patterns based on DHDH levels. Hierarchical clustering stratified by DHDH expression identified significant transcriptomic differences between the high- and low-expression groups (**Figure 1C**). Gene Ontology (GO) enrichment analysis of DHDH-co-expressed genes indicated significant enrichment in biological processes such as chromosome segregation, spindle organization, and mitotic nuclear division (**Figure 1D**), suggesting a potential role of DHDH in cell cycle regulation.

To explore the relationship between DHDH and the tumor immune microenvironment, we assessed immune cell infiltration levels across different DHDH expression groups. High DHDH expression was associated with a significantly reduced infiltration of CD8^+^ T cells, activated dendritic cells (aDCs), macrophages, and natural killer (NK) cells, along with an increase in regulatory T cells (Tregs) (**Figure 1E**). Notably, the enrichment score of CD8^+^ T cells was significantly lower in the DHDH-high group (**Figure 1F**), indicating a potential link between DHDH overexpression and immunosuppressive phenotype. Furthermore, key immune checkpoint molecules, including PDCD1 (PD-1), CD274 (PD-L1), CTLA4, and LAG3, were significantly upregulated in the DHDH-high group (**Figure 1G**). Correlation analysis revealed a modest but statistically significant positive association between DHDH and CD274 expression (Spearman r = 0.15, *P* = 0.0027; **Figure 1H**). Although the correlation coefficient was relatively small, this finding indicates a weak positive relationship between DHDH expression and CD274 levels in HCC.

### 3.2 High DHDH expression is associated with poor prognosis in HCC

**Figure 2:**
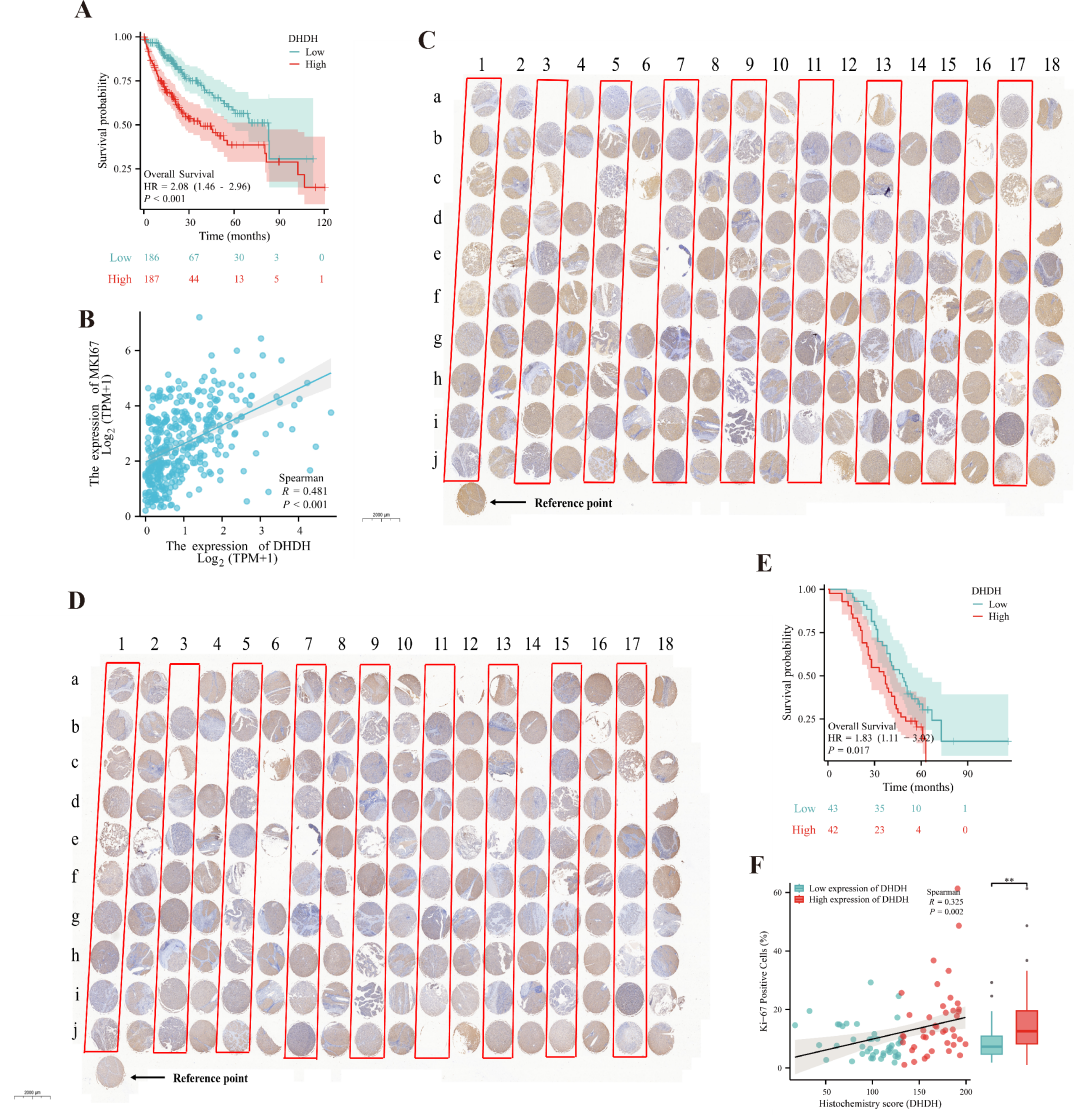
High DHDH expression is associated with poor prognosis and increased tumor cell proliferation in HCC. **(A, B)** Analysis of the TCGA-LIHC cohort shows that high DHDH expression is significantly associated with reduced overall survival (OS) **(A)** and positively correlated with the expression of the proliferation marker Ki-67 **(B)**. **(C-F)** HCC tissue microarray analysis further validates these associations. Representative IHC images of DHDH **(C)** and Ki-67 **(D)** staining are shown. Samples were stratified into high and low DHDH expression groups based on H-score. Survival analysis indicates worse prognosis in the DHDH-high group **(E)**, which also exhibited a significantly higher proportion of Ki-67-positive cells. Correlation analysis confirms a positive association between DHDH expression and the Ki-67-positive cell ratio **(F)**.

Given the observed upregulation of DHDH and its association with an immunosuppressive microenvironment in HCC, we next investigated its clinical prognostic significance and correlation with tumor cell proliferation. Kaplan-Meier survival analysis based on the TCGA-LIHC cohort revealed that patients with high DHDH expression had significantly shorter overall survival (OS) than those with low expression (HR = 2.08, 95% CI: 1.46-2.96, *P* < 0.001; **Figure 2A**). In addition, DHDH expression was positively correlated with the proliferation marker Ki-67, a clinically relevant indicator of malignancy and prognosis (Spearman *R* = 0.481, *P* < 0.001; **Figure 2B**).

To validate these findings, we assessed DHDH expression in HCC tissue microarrays (**Figure 2C**) and stratified the samples into high and low expression groups based on the H-score. Comparative analysis showed that the proportion of Ki-67-positive cells was significantly higher in the DHDH-high group (*P* = 0.001; **Figures 2D, F**), suggesting a link between elevated DHDH expression and enhanced proliferative activity in HCC. Kaplan-Meier analysis based on follow-up data from the tissue microarray cohort also confirmed that patients with high DHDH expression had worse OS (HR = 1.83, 95% CI: 1.11-3.02, *P* = 0.017; **Figure 2E**). Correlation analysis further supported a positive association between DHDH expression and the proportion of Ki-67-positive cells (Spearman’s *R* = 0.325, *P* = 0.002; **Figure 2F**).

The baseline clinical characteristics were compared between the groups (**Supplementary Tables 1**, **2**). In TCGA cohort, patients in the DHDH-high group had significantly more advanced tumor stages, higher rates of vascular invasion, and elevated alpha-fetoprotein (AFP) levels (*P* < 0.05). In the tissue microarray cohort, high DHDH expression was associated with older age (*P* < 0.001) and increased AFP levels (*P* = 0.029), further supporting its role as a potentially unfavorable prognostic marker.

Cox regression analysis was performed to evaluate the prognostic relevance of DHDH. In univariate analysis, high DHDH expression was significantly associated with poor survival (hazard ratio [HR] = 2.076, 95% confidence interval [CI]: 1.455-2.963, *P* < 0.001). Importantly, multivariate analysis adjusted for clinical variables such as age, sex, and tumor stage confirmed that DHDH overexpression remained an independent predictor of poor prognosis in HCC (HR = 1.834, 95% CI: 1.151-2.923, *P* = 0.011; **Supplementary Table 4**). These findings further underscore the potential of DHDH as a prognostic biomarker of HCC.

### 3.3 DHDH regulates HCC cell proliferation, migration, invasion, and CD8^+^ T cell function

To investigate the functional role of DHDH in HCC, we established Hepa1–6 cell models with stable DHDH overexpression (oeDHDH) and knockdown (shDHDH). Fluorescence imaging confirmed successful model construction (**Supplementary Figure 1A**), and both qPCR and western blot analyses verified that DHDH expression was significantly upregulated in the oeDHDH group and downregulated in the shDHDH group (**Supplementary Figures 1B, C**; **3A, B**).

**Figure 3:**
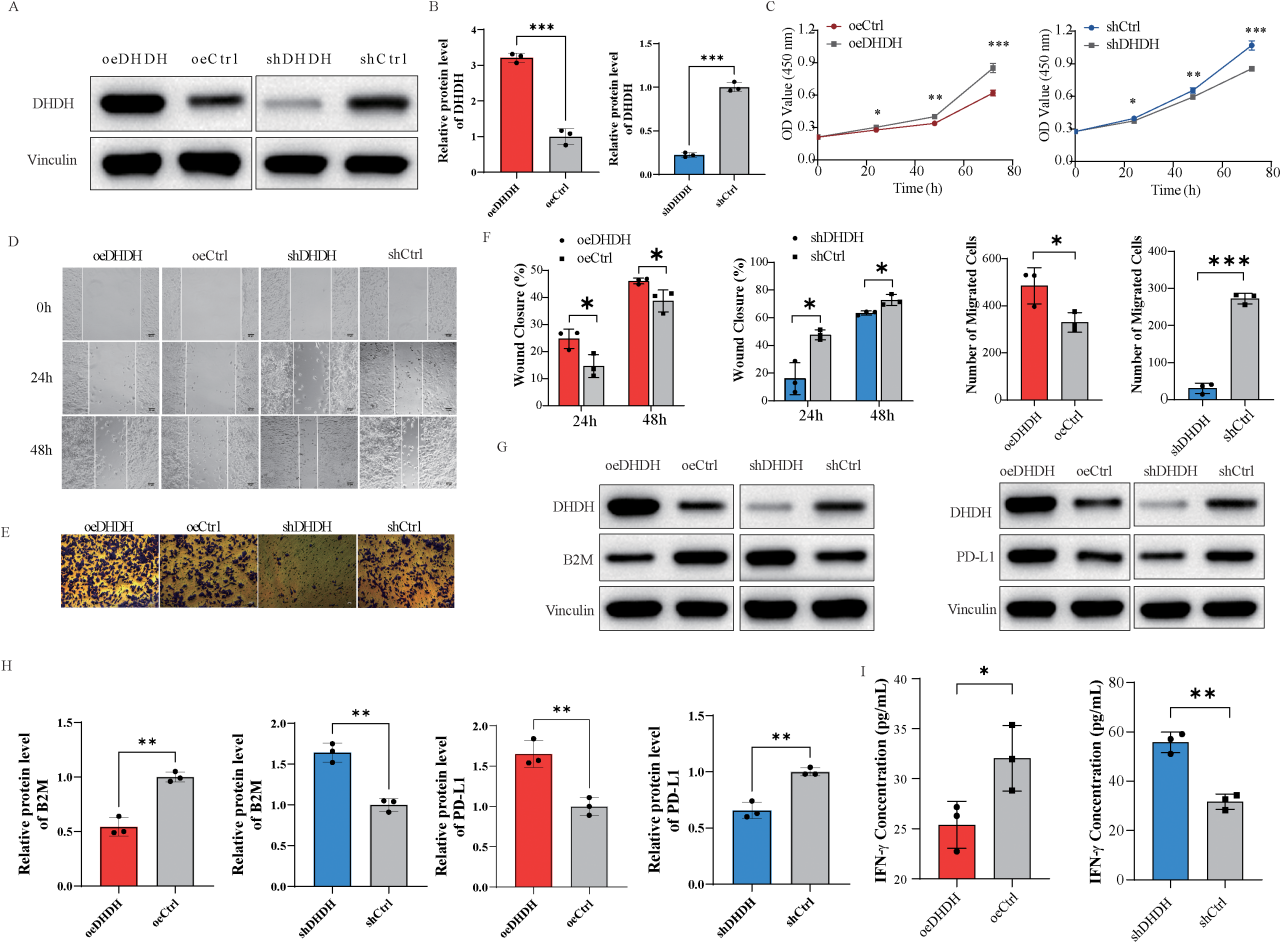
DHDH regulates HCC cell proliferation, migration, invasion, and the immune microenvironment. **(A, B)** Western blot analysis of DHDH protein expression levels in Hepa1–6 cells with DHDH overexpression (oeDHDH) or knockdown (shDHDH). **(C)** Cell proliferation assessed by CCK-8 assay. **(D, F)** Scratch wound healing assay and quantitative analysis showing that DHDH enhances cell migration. **(E, F)** Transwell invasion assay and quantification indicating that DHDH promotes invasive capacity. **(G, H)** Western blot analysis showing increased PD-L1 and decreased B2M expression in oeDHDH cells and the opposite in shDHDH cells. **(I)** ELISA measurement of IFN-γ secretion from CD8^+^ T cells, demonstrating that DHDH impairs T cell effector function. * represents P < 0.05; ** represents P < 0.01; *** represents P < 0.001.

Functional assays demonstrated that DHDH enhances the proliferative capacity of HCC cells. CCK-8 assays revealed significantly accelerated cell proliferation in the oeDHDH group, whereas the shDHDH group showed suppressed proliferation (**Figure 3C**). Wound healing assays indicated that DHDH overexpression markedly enhanced by DHDH migration, which was attenuated by DHDH knockdown (**Figures 3D, F**). Similarly, Transwell invasion assays demonstrated that DHDH promoted invasive potential, with oeDHDH cells showing increased invasion and shDHDH cells showing reduced invasion (**Figures 3E, F**). Flow cytometric analysis of the cell cycle revealed a decreased G1 phase proportion and an increased S phase proportion in the oeDHDH group, whereas the shDHDH group exhibited the opposite trend (**Supplementary Figures 1D–F**), suggesting that DHDH promotes cell cycle progression and proliferative activity in HCC cells.

Regarding immune-related mechanisms, western blot analysis showed that DHDH overexpression led to the upregulation of programmed death-ligand 1 (PD-L1) and downregulation of B2M, while DHDH knockdown had the opposite effect (**Figures 3G, H**). CD8^+^ T cells were isolated using a negative selection kit (**Supplementary Figure 1G**) and co-cultured with tumor cells. Co-culture assays revealed that CD8^+^ T cells secreted significantly lower levels of interferon-gamma (IFN-γ) in the oeDHDH group, whereas IFN-γ secretion was significantly increased in the shDHDH group (**Figure 3I**).

Collectively, these results demonstrate that DHDH promotes HCC cell proliferation, migration, and invasion, while modulating PD-L1 and B2M expression to suppress CD8^+^ T cell effector function, thereby contributing to immune evasion within the HCC tumor microenvironment.

### 3.4 DHDH promotes tumor growth and modulates the immune microenvironment in HCC

**Figure 4:**
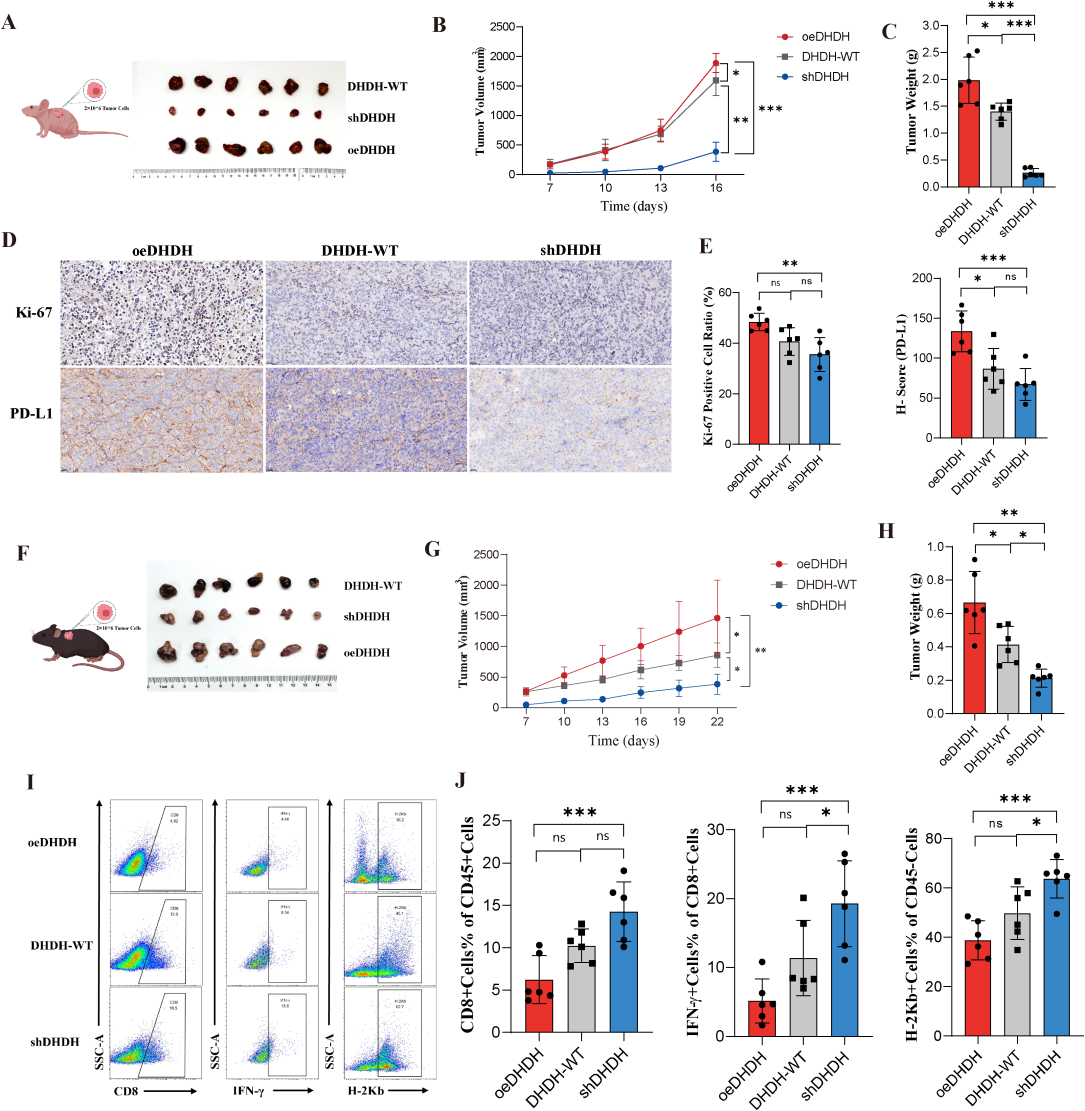
DHDH regulates tumor growth and the immune microenvironment in HCC. **(A-C)** Subcutaneous tumor models in BALB/c nude mice showed that tumors in the oeDHDH group exhibited significantly increased volume and weight, whereas tumor growth was markedly attenuated in the shDHDH group. **(D, E)** Immunohistochemical staining of tumor tissues revealed elevated expression of Ki-67 and PD-L1 in the oeDHDH group and decreased levels in the shDHDH group. **(F-H)** In C57BL/6 immunocompetent mice, the oeDHDH group developed larger tumors, while the shDHDH group displayed suppressed tumor growth. **(I, J)** Flow cytometric analysis of tumor-infiltrating immune cells showed that the oeDHDH group had reduced proportions of CD8^+^ T cells, IFN-γ^+^ CD8^+^ T cells, and H-2Kb^+^ cells, whereas these populations were increased in the shDHDH group. * represents P < 0.05; ** represents P < 0.01; *** represents P < 0.001.

To further investigate the biological role of DHDH *in vivo*, we established subcutaneous tumor models using different Hepa1–6 cell lines in immunodeficient BALB/c nude mice. Tumors derived from the oeDHDH group exhibited significantly greater volume and weight than those from the DHDH-WT group, while tumor growth in the shDHDH group was markedly suppressed (**Figures 4A–C**). Immunohistochemical analysis revealed increased expression of the proliferation marker Ki-67 and immune checkpoint molecule PD-L1 in tumors from the oeDHDH group, whereas their expression was significantly reduced in the shDHDH group (**Figures 4D, E**; **Supplementary Figures 2A–C**).

In immunocompetent C57BL/6 mice, similar trends were observed: both tumor volume and weight were significantly increased in the oeDHDH group and suppressed in the shDHDH group (**Figures 4F–H**). Flow cytometry analysis of tumor-infiltrating immune cells (gating strategy shown in **Supplementary Figure 2F**) revealed a substantial decrease in the proportion of CD8^+^ T cells, IFN-γ-producing CD8^+^ T cells, and H-2Kb^+^ antigen-presenting cells in the oeDHDH group. In contrast, the shDHDH group exhibited an increased frequencies of CD8^+^ T cells and their functional subsets (**Figures 4I, J**).

Collectively, these findings demonstrated that DHDH promotes tumor growth in both immunodeficient and immunocompetent hosts, likely by reshaping the immune microenvironment and suppressing CD8^+^ T cell-mediated antitumor immunity, thereby facilitating HCC progression.

### 3.5 D-xylose suppresses HCC tumor growth and enhances the efficacy of anti-PD-L1 therapy by modulating the immune microenvironment

**Figure 5:**
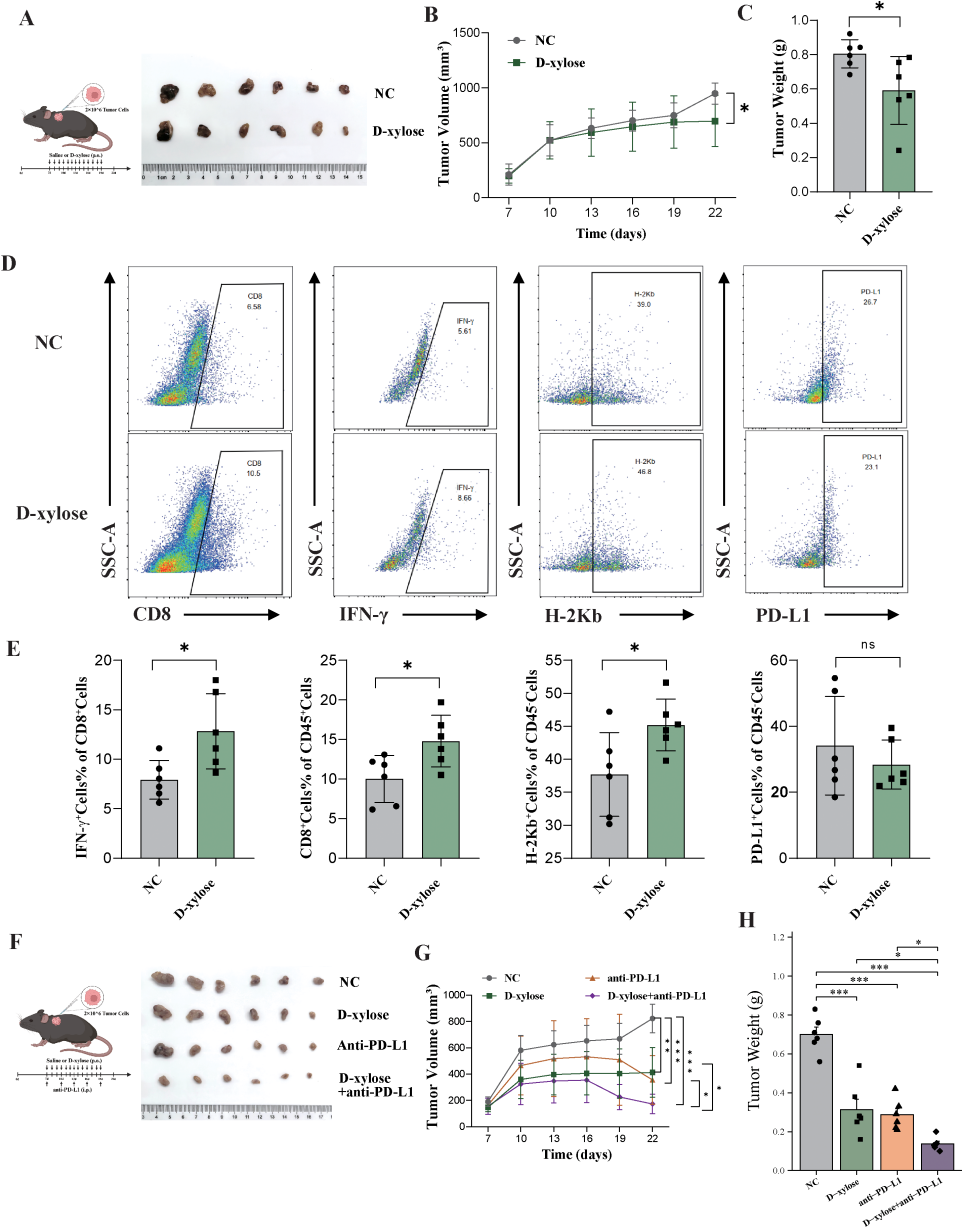
D-xylose suppresses HCC tumor growth and enhances the efficacy of anti-PD-L1 therapy. **(A-C)** Subcutaneous tumor model using Hepa1–6 cells in C57BL/6 mice showed that D-xylose treatment significantly reduced tumor volume and weight compared to the control group. **(D, E)** Flow cytometry analysis of tumor-infiltrating immune cells revealed increased proportions of CD8^+^ T cells, IFN-γ^+^ CD8^+^ T cells, and H-2Kb^+^ cells, along with decreased PD-L1^+^ cells in the D-xylose treatment group. **(F-H)** Combination therapy with D-xylose and anti-PD-L1 antibody further enhanced the antitumor effect, with the lowest tumor volume and weight observed in the combination group compared to either monotherapy. * represents P < 0.05; *** represents P < 0.001.

Based on data from the NCBI database, DHDH is recognized as a key enzyme involved in D-xylose metabolism. Therefore, we hypothesized that D-xylose may modulate the tumor microenvironment and antitumor immune responses in HCC by interfering with the DHDH-mediated metabolic pathways. To test this hypothesis, we evaluated the immunomodulatory and tumor-inhibitory effects of D-xylose in a subcutaneous tumor model using oeDHDH-expressing Hepa1–6 cells in immunocompetent C57BL/6 mice. Tumor volume and weight were significantly reduced in mice treated with D-xylose compared with those in the negative control (NC) group (**Figures 5A-C**). Flow cytometric analysis of tumor-infiltrating immune cells revealed that D-xylose treatment significantly increased the proportion of CD8^+^ T cells, IFN-γ^+^ CD8^+^ T cells, and H-2Kb^+^ cells, while decreasing the proportion of PD-L1^+^ cells (**Figures 5D, E**). Histological evaluation of the tumor tissue architecture was performed using hematoxylin and eosin (H&E) staining (**Supplementary Figure 2D**).

To further explore the therapeutic potential of D-xylose in combination with immune checkpoint blockade, we treated oeDHDH tumor-bearing mice with D-xylose, anti-PD-L1 antibody, or their combination. Although both monotherapies led to tumor growth inhibition, the combination treatment produced the most pronounced reduction in tumor volume and weight (**Figures 5F-H**), suggesting a synergistic antitumor effect. H&E staining was also conducted to assess tumor tissue architecture in the combination therapy setting (**Supplementary Figure 2E**). Taken together, these results demonstrate that D-xylose not only enhances CD8^+^ T cell-mediated immune responses within the tumor microenvironment but also synergizes with PD-L1 blockade to potentiate antitumor efficacy.

### 3.6 DHDH drives immune evasion in HCC organoids, while D-xylose remodels the microenvironment and enhances immunotherapeutic response

**Figure 6:**
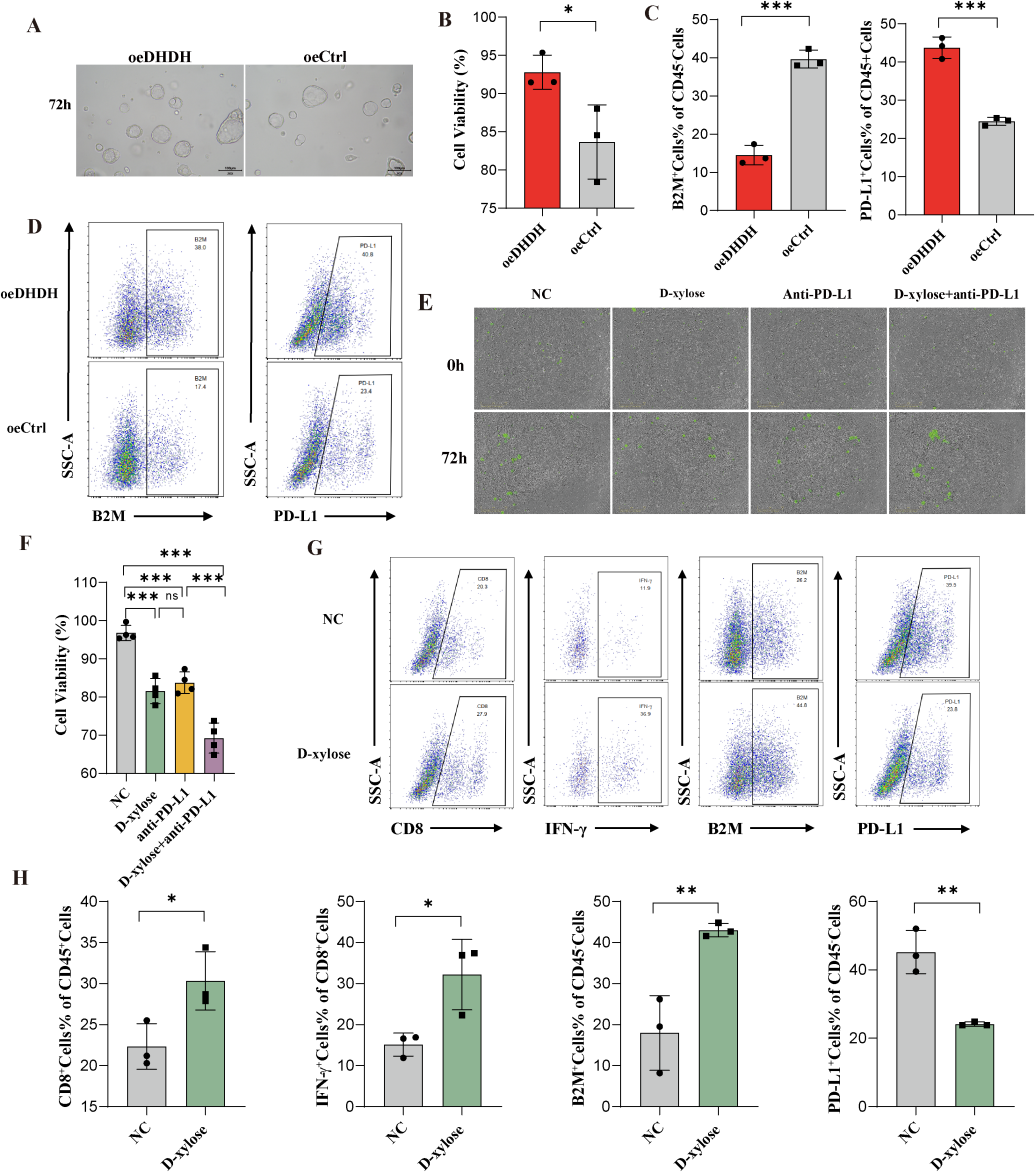
DHDH promotes an immunosuppressive phenotype in HCC organoids, while D-xylose enhances their responsiveness to immunotherapy. **(A, B)** Three-dimensional organoid culture shows that oeDHDH organoids exhibit denser structures and significantly increased cellular viability. **(C, D)** Flow cytometric analysis reveals elevated PD-L1 expression and decreased B2M expression in oeDHDH organoids. **(E, F)** In organoid-PBMC co-culture models, combined treatment with D-xylose and anti-PD-L1 antibody significantly reduces organoid viability compared to monotherapies. **(G, H)** Flow cytometry of co-cultures demonstrates that D-xylose increases CD8^+^ T cell infiltration and IFN-γ production, enhances B2M expression, and reduces PD-L1 expression, indicating a shift in the tumor immune microenvironment from suppressive to activated. * represents P < 0.05; ** represents P < 0.01; *** represents P < 0.001.

To evaluate the function of DHDH in a more physiologically relevant model, we established DHDH-overexpressing hepatocellular carcinoma (HCC) organoids (oeDHDH). Morphologically, oeDHDH organoids exhibited denser and more proliferative growth than the control group (oeCtrl) after 72 h of culture (**Figure 6A**). Quantitative analysis confirmed a significant increase in cell viability in the oeDHDH group (**Figure 6B**).

Flow cytometric analysis was conducted to assess the expression of surface molecules associated with immune modulation in the HCC organoids. Compared with the controls, oeDHDH organoids showed markedly reduced expression of B2M and significantly elevated levels of PD-L1 (**Figures 6C, D**), indicating that DHDH overexpression induces an immunosuppressive phenotype.

To further investigate therapeutic implications, organoids were co-cultured with peripheral blood mononuclear cells (PBMCs) and subjected to various treatments. After 72 h, both D-xylose and anti-PD-L1 monotherapy moderately reduced organoid viability; however, the combination treatment led to significantly greater suppression of organoid activity (**Figure 6E**). Quantitative analysis confirmed that the cytotoxic effect of the combination treatment was superior to that of either treatment alone (**Figure 6F**).

Flow cytometric evaluation of the co-cultures revealed that D-xylose treatment significantly increased the proportion of infiltrating CD8^+^ T cells and enhanced their IFN-γ secretion (**Figures 6G, H**, left panels), suggesting improved T cell functionality. Concurrently, D-xylose upregulated MHC class I molecule (B2M) expression and downregulated PD-L1 levels in tumor cells (**Figures 6G, H**, right panels), indicating that D-xylose effectively reversed the immunosuppressive tumor microenvironment.

### 3.7 D-xylose modulates the NADP^+^/NADPH redox balance to reshape the ROS-mediated oxidative microenvironment and improve T cell function

**Figure 7:**
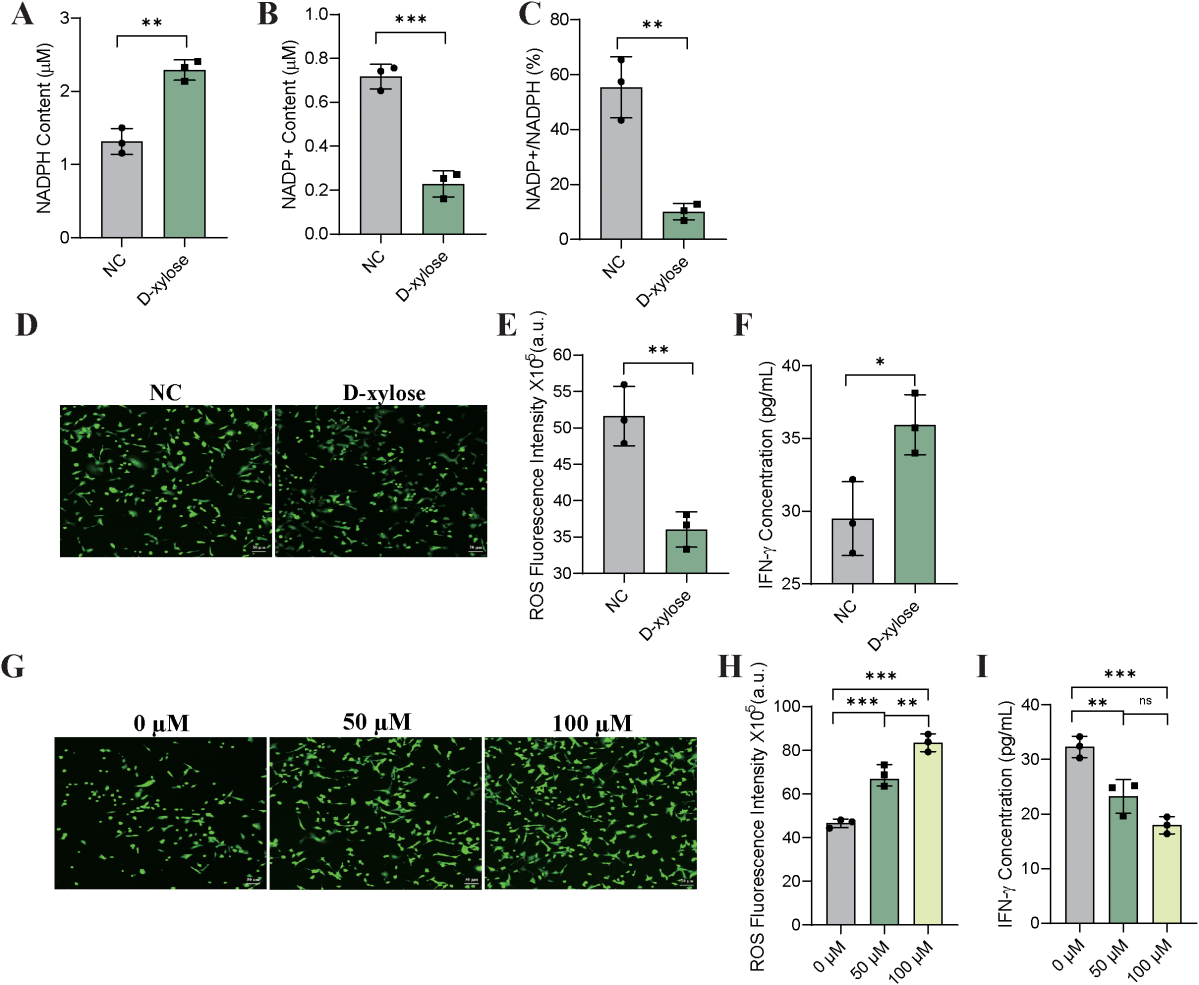
D-xylose enhances NADPH production via DHDH metabolic activity and alleviates ROS-mediated T cell dysfunction. **(A-C)** Quantitative analysis of intracellular NADPH levels **(A)**, NADP^+^ levels **(B)**, and NADP^+^/NADPH ratio **(C)** in oeDHDH cells treated with or without D-xylose (200 μM for 24 h). **(D-F)** Effects of D-xylose on redox status and CD8^+^ T cell functionality. **(D)** Representative fluorescence microscopy images of ROS levels after staining with DCFH-DA (illustrative only). **(E)** Quantification of ROS levels using a fluorescence microplate reader. **(F)** ELISA-based measurement of IFN-γ secretion by CD8^+^ T cells co-cultured with oeDHDH cells in the presence or absence of D-xylose. **(G-I)** Effects of H_2_O_2_-induced ROS elevation on CD8^+^ T cell function. **(G)** Representative ROS fluorescence images of oeDHDH cells treated with different concentrations of H_2_O_2_ (0, 50, 100 μM). **(H)** Quantitative measurement of ROS levels by using a microplate reader. **(I)** IFN-γ secretion by CD8^+^ T cells co-cultured with oeDHDH cells at different H_2_O_2_ concentrations as assessed by ELISA.

To determine whether D-xylose regulates the intracellular redox state and T cell functionality through the modulation of DHDH metabolic activity, we assessed the levels of NADPH, NADP^+^, and their ratio in oeDHDH cells following D-xylose treatment. The results showed that D-xylose markedly increased intracellular NADPH levels (**Figure 7A**), decreased NADP^+^ levels (**Figure 7B**), and reduced the NADP^+^/NADPH ratio (**Figure 7C**), indicating a shift toward a more reduced cellular environment.

Representative fluorescence images of ROS staining are shown for illustrative purposes (**Figure 7D**), whereas quantitative ROS analysis using a fluorescence microplate reader revealed a significant decrease in ROS levels upon D-xylose treatment (**Figure 7E**). In the co-culture experiments, D-xylose treatment significantly enhanced IFN-γ secretion by CD8^+^ T cells (**Figure 7F**), suggesting improved T cell function.

To further assess the impact of ROS on T cell activity, oeDHDH cells were treated with different concentrations of hydrogen peroxide (H_2_O_2_). Representative ROS fluorescence images (**Figure 7G**) and the corresponding quantitative analysis (**Figure 7H**) confirmed a dose-dependent increase in ROS levels. When these cells were co-cultured with CD8^+^ T cells, elevated ROS levels were associated with a significant reduction in IFN-γ production (**Figure 7I**). These findings suggest that enhancing NADPH synthesis via D-xylose can attenuate oxidative stress, thereby alleviating ROS-induced T-cell suppression and restoring antitumor immune function.

## 4 Discussion

HCC is one of the most lethal malignancies globally, and its progression is driven not only by the intrinsic proliferative and metastatic capacities of tumor cells but also by the liver-specific immune microenvironment (1, 2, 20, 21). Although ICIs have yielded notable therapeutic advances in HCC, the overall response rate remains limited owing to the complexity of the tumor microenvironment and mechanisms of immune evasion (3, 21). Therefore, a deeper understanding of the crosstalk between metabolic reprogramming and immune regulation in HCC is essential for the development of next-generation anti-tumor strategies. In this study, we systematically elucidated the role of DHDH in modulating the metabolism-immunity axis in HCC and demonstrated that D-xylose, as a competitive substrate of DHDH, can reshape the tumor immune microenvironment by altering the NADP^+^/NADPH balance. This remodeling enhances CD8^+^ T cell function and, when combined with anti-PD-L1 therapy, produces synergistic antitumor effects. These findings not only deepen the understanding of HCC metabolic-immune regulation but also provide a theoretical rationale for the development of combined metabolic and immunotherapeutic approaches.

DHDH is an NAD(P)^+^-dependent oxidoreductase overexpressed in various cancers. Previous studies on TNBC have shown that DHDH suppresses the expression of the immunoproteasome subunit PSMB9 by modulating D-xylose metabolism, leading to defective antigen processing and immune evasion (18). Here, we report for the first time that DHDH is markedly upregulated in HCC and is not only closely associated with tumor cell proliferation, but also with a profoundly immunosuppressive microenvironment. Specifically, high DHDH expression correlated with reduced CD8^+^ T cell infiltration, upregulation of PD-L1, and downregulation of B2M, indicating impaired antigen presentation. Although the correlation between DHDH and PD-L1 in the TCGA dataset was modest in magnitude, our experimental findings consistently demonstrated an association between DHDH expression and PD-L1 regulation. Functional assays further confirmed that DHDH promoted HCC cell proliferation, migration, and invasion, while inhibiting CD8^+^ T cell-mediated cytotoxic responses. These findings underscore that DHDH is a key metabolic-immune regulator in the HCC tumor microenvironment.

Recent studies have demonstrated that the downregulation of B2M not only compromises the stability of the MHC class I complex but also reflects dysfunction of its upstream transcriptional regulator, NLRC5. NLRC5 also known as the class I transactivator (CITA), coordinately regulates the expression of H-2Kb, B2M, TAP1/2, and immunoproteasome subunits, thereby determining the ability of tumor cells to be recognized by CD8^+^ T cells. Loss or suppression of NLRC5 has been identified as a critical mechanism of immune evasion and resistance to immune checkpoint blockade across multiple tumor types (22, 23). Consistent with these findings, our data showed that DHDH overexpression led to decreased B2M expression and reduced H-2Kb^+^ antigen-presenting cells, suggesting that DHDH-mediated metabolic reprogramming may suppress the NLRC5-driven transcriptional network, thereby further attenuating MHC-I expression and antigen presentation. Considering that the IFN-γ/STAT1–IRF1–NLRC5 axis represents a canonical pathway for MHC-I induction (22–24), the NADPH/ROS imbalance caused by DHDH overexpression may interfere with this signaling cascade, thus linking metabolic perturbation to immune evasion. This hypothesis provides a novel mechanistic perspective on how DHDH contributes to immune suppression in HCC.

Contrary to the traditional view that NADPH accumulation primarily supports the antioxidant defense of tumor cells (25, 26), our study revealed that D-xylose competitively interferes with DHDH-mediated metabolism, leading to a significant elevation in NADPH levels, which in turn exerts a favorable immunomodulatory effect within the tumor microenvironment. Rather than conferring a growth advantage to tumor cells, increased NADPH promoted the restoration of antigen presentation, downregulation of PD-L1, and enhancement of CD8^+^ T cell function. These findings suggest that, in specific metabolic contexts, NADPH accumulation may play a non-canonical role in immune regulation. Previous studies have demonstrated that NADPH is critical for maintaining redox homeostasis in T cells and is essential for preventing T cell dysfunction in the ROS-rich tumor microenvironment (27, 28). Thus, D-xylose may regulate NADPH availability via DHDH, alleviating oxidative stress in the tumor microenvironment (TME), while preferentially supporting the metabolic adaptability of effector T cells (Teff), thereby restoring their antitumor activity. Recent evidence indicates that the dynamic balance between NADPH and ROS is a key determinant of the immune status in the TME, and its disruption compromises antigen presentation and T cell cytotoxicity (29, 30). These findings challenge the conventional paradigm that NADPH accumulation facilitates tumor progression and instead highlight its complex bidirectional role in metabolism-immune crosstalk under specific microenvironmental conditions.

In animal models, treatment with D-xylose significantly inhibited HCC tumor growth. Flow cytometry revealed increased intratumoral infiltration of CD8^+^ T cells, elevated IFN-γ secretion, and upregulated H-2Kb expression, suggesting enhanced antigen presentation. Moreover, combination therapy with anti-PD-L1 antibody further augmented the antitumor effect. This “metabolism plus immunotherapy” combinatorial strategy aligns well with the emerging concept of TME remodeling (31). For instance, metabolic reprogramming of tumor-associated macrophages (TAMs) to induce M2-to-M1 repolarization facilitates antigen presentation and enhances CD8^+^ T-cell activity (32). Similarly, targeting the Nrf2 pathway disrupts redox homeostasis, promotes immunogenic cell death (ICD), and synergizes with T cell activation, offering promising therapeutic outcomes (33). These studies underscore the importance of targeting the metabolic-immune crosstalk to improve ICI efficacy.

Consistently, similar expression trends were observed in patient-derived HCC organoids, further confirming the regulatory role of DHDH in antigen presentation and immune modulation at the organoid level. As three-dimensional models that faithfully recapitulate the architecture and molecular features of primary tumors, organoids have garnered increasing attention for their ability to retain intratumoral heterogeneity and preserve cell-cell interactions, thereby offering unique advantages for mechanistic studies and translational research (34, 35). However, their dense structure, cellular polarity, and dependence on the extracellular matrix pose significant challenges for gene transfection compared with conventional two-dimensional cell lines (36). In this study, we established stable DHDH expression in HCC organoids by optimizing the transfection protocol, thus providing a reliable tool for functional investigation. Moreover, the co-culture system of HCC organoids with PBMCs introduced immune components into the *in vitro* model, enabling a more physiologically relevant simulation of metabolic-immune interactions within the TME. This approach further validated the pivotal role of DHDH in modulating T-cell function, PD-L1 expression, and antigen presentation pathways at the tissue level. Overall, the integration of patient-derived organoids and immune co-culture not only reinforces the robustness of our findings but also highlights their clinical relevance and translational potential.

Despite the comprehensive elucidation of the role of DHDH in HCC, this study has several limitations. First, the specific regulatory mechanisms by which NADPH accumulation modulates distinct immune cell subsets are not fully understood. In particular, whether NADPH enhances the TME by influencing dendritic cell (DC)-mediated cross-presentation or reprogramming the metabolic state of TAMs warrants further investigation. Second, the pharmacokinetics, long-term safety, and optimal dosing and timing of D-xylose in combination with ICIs require systematic evaluation. Future studies should explore the synergistic interactions between DHDH and other key metabolic enzymes, such as G6PD and MTHFD2, and focus on the development of highly specific DHDH inhibitors via nanocarrier-based delivery systems to achieve precise, low-toxicity therapeutic interventions with translational potential.

In summary, this study is the first to identify the central role of DHDH in the metabolic-immune regulation of HCC and reveals a novel mechanism by which D-xylose modulates the TME through NADPH and enhances immunotherapeutic responses. These findings not only deepen our understanding of metabolism-immunity crosstalk in HCC, but also provide robust experimental support for the development of next-generation “metabolism plus immunity” combination therapies.

## Data Availability

The original contributions presented in the study are included in the article/**Supplementary Material**. Further inquiries can be directed to the corresponding authors.
