# Supplementary material for: D-xylose suppresses hepatocellular carcinoma progression by regulating dihydrodiol dehydrogenase and remodeling the immune microenvironment
Source: Front Immunol. 2026 Mar 13;17:1792196. doi: 10.3389/fimmu.2026.1792196 (PMC13021656; doi:10.3389/fimmu.2026.1792196)
Supplement: Supplementary file 1 [file DataSheet1.docx]

Due to the large file sizes of the HE and IHC images, these images are not included in this folder and have instead been uploaded to the Jianguoyun cloud platform **(URL: https://www.jianguoyun.com/p/Da7x50gQh82ADhiexpsGIAA).**
