# Supplementary figures and images for: D-xylose suppresses hepatocellular carcinoma progression by regulating dihydrodiol dehydrogenase and remodeling the immune microenvironment
Source: Front Immunol. 2026 Mar 13;17:1792196. doi: 10.3389/fimmu.2026.1792196 (PMC13021656; doi:10.3389/fimmu.2026.1792196)

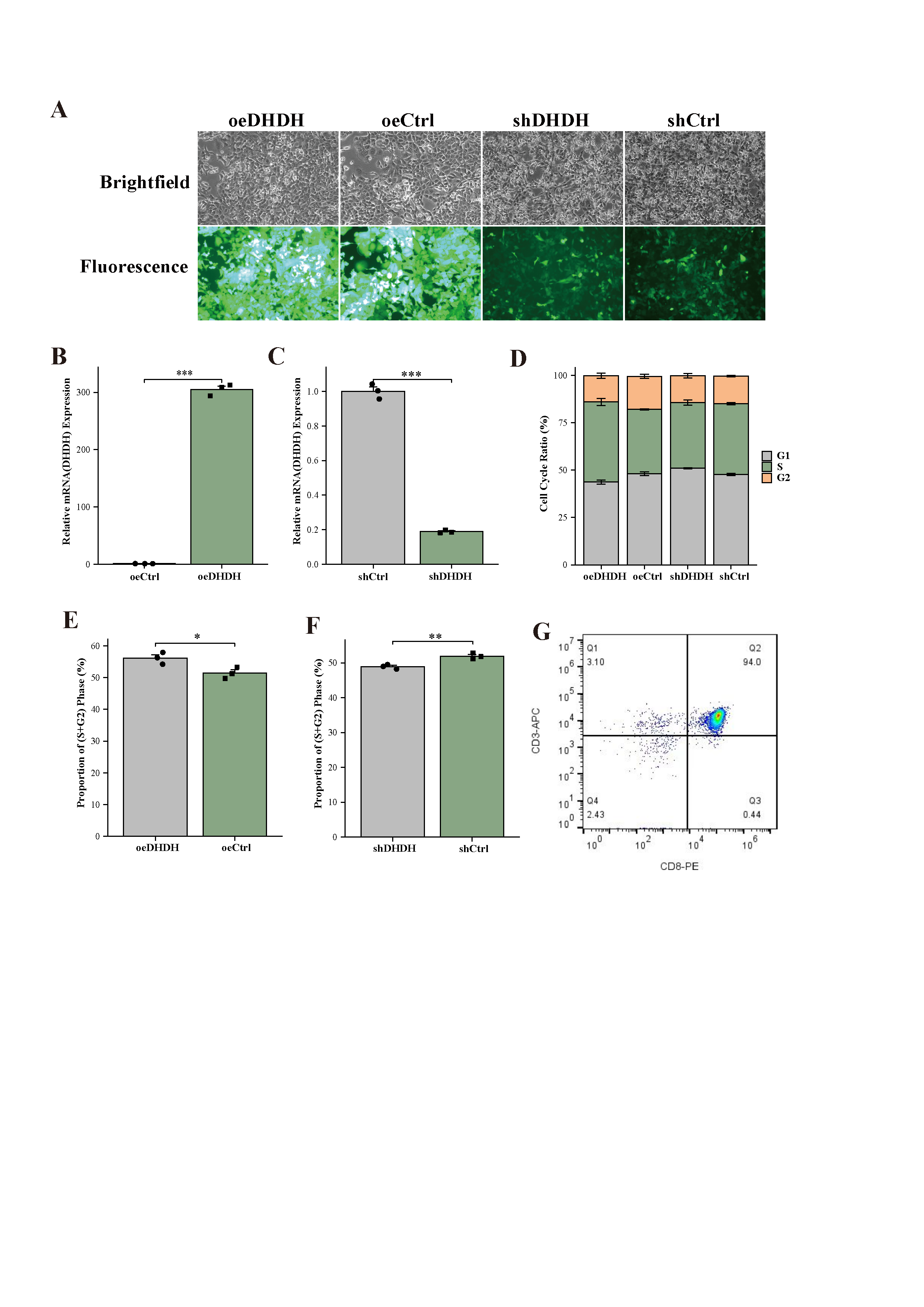

Supplement: Supplementary file 2 [file Image1.tif]

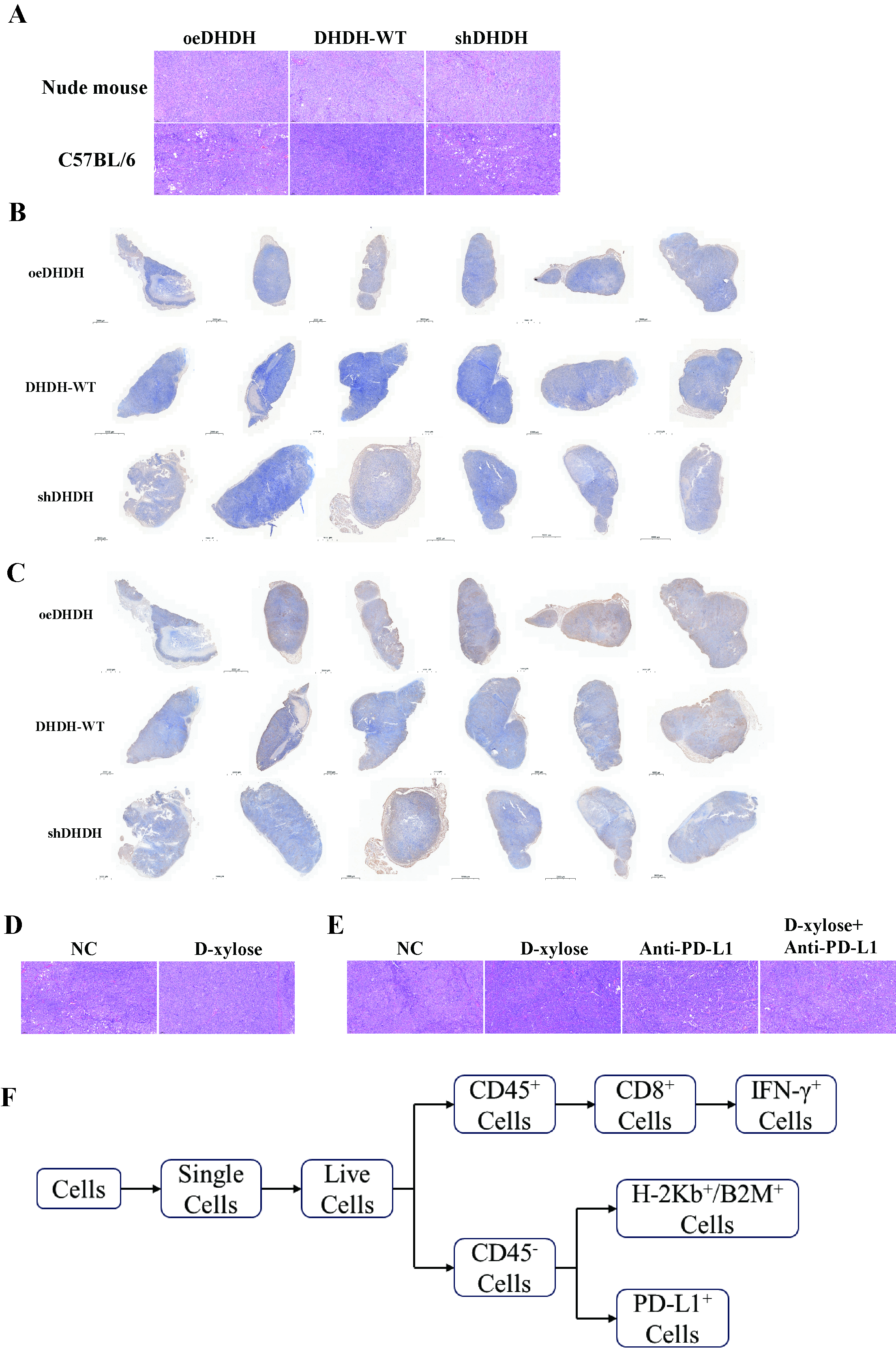

Supplement: Supplementary file 3 [file Image2.tif]

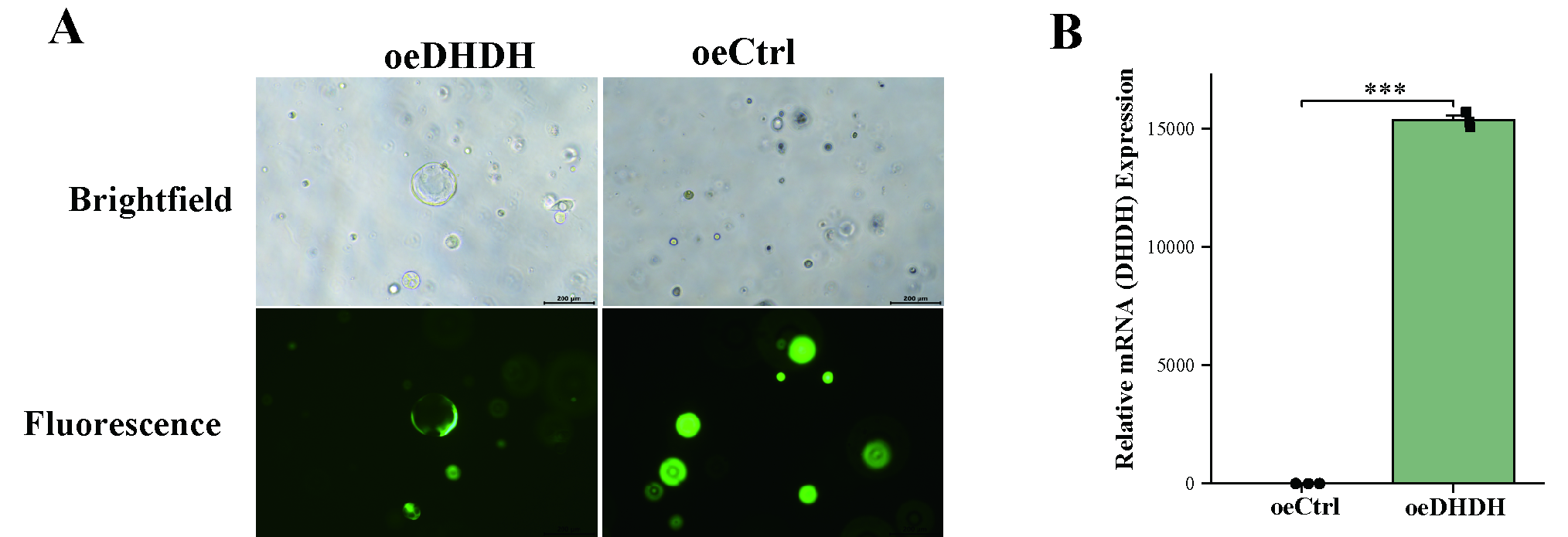

Supplement: Supplementary file 4 [file Image3.tif]
