# Supplementary material for: D-xylose suppresses hepatocellular carcinoma progression by regulating dihydrodiol dehydrogenase and remodeling the immune microenvironment
Source: Front Immunol. 2026 Mar 13;17:1792196. doi: 10.3389/fimmu.2026.1792196 (PMC13021656; doi:10.3389/fimmu.2026.1792196)
Supplement: Supplementary file 5 [file Table1.docx]

**Table S1. Clinical and pathological characteristics of HCC patients from the TCGA cohort**

| Characteristics | Low expression of DHDH (n=187) | High expression of DHDH (n=187) | *P* value |
| --- | --- | --- | --- |
| Gender, n (%) |  |  | 0.097 |
| Male | 134 (35.8%) | 119 (31.8%) |  |
| Female | 53 (14.2%) | 68 (18.2%) |  |
| Age, n (%) |  |  | 0.275 |
| <= 60 | 83 (22.3%) | 94 (25.2%) |  |
| > 60 | 103 (27.6%) | 93 (24.9%) |  |
| Race, n (%) |  |  | 0.024 |
| Asian | 68 (18.8%) | 92 (25.4%) |  |
| Black or African American | 7 (1.9%) | 10 (2.8%) |  |
| White | 105 (29%) | 80 (22.1%) |  |
| Pathologic T stage, n (%) |  |  | **< 0.001** |
| T1 | 111 (29.9%) | 72 (19.4%) |  |
| T2 | 36 (9.7%) | 59 (15.9%) |  |
| T3 | 33 (8.9%) | 47 (12.7%) |  |
| T4 | 4 (1.1%) | 9 (2.4%) |  |
| Pathologic N stage, n (%) |  |  | 0.636 |
| N0 | 126 (48.8%) | 128 (49.6%) |  |
| N1 | 1 (0.4%) | 3 (1.2%) |  |
| Pathologic M stage, n (%) |  |  | 1.000 |
| M0 | 127 (46.7%) | 141 (51.8%) |  |
| M1 | 2 (0.7%) | 2 (0.7%) |  |
| Pathologic stage, n (%) |  |  | **0.001** |
| Stage I | 105 (30%) | 68 (19.4%) |  |
| Stage II | 35 (10%) | 52 (14.9%) |  |
| Stage III | 33 (9.4%) | 52 (14.9%) |  |
| Stage IV | 3 (0.9%) | 2 (0.6%) |  |
| Tumor status, n (%) |  |  | 0.443 |
| Tumor free | 106 (29.9%) | 96 (27%) |  |
| With tumor | 74 (20.8%) | 79 (22.3%) |  |
| Histological type, n (%) |  |  | 0.207 |
| Fibrolamellar carcinoma | 3 (0.8%) | 0 (0%) |  |
| Hepatocellular carcinoma | 181 (48.4%) | 183 (48.9%) |  |
| Hepatocholangiocarcinoma (mixed) | 3 (0.8%) | 4 (1.1%) |  |
| Child-Pugh grade, n (%) |  |  | 0.306 |
| A | 117 (48.5%) | 102 (42.3%) |  |
| B | 14 (5.8%) | 7 (2.9%) |  |
| C | 1 (0.4%) | 0 (0%) |  |
| AFP(ng/ml), n (%) |  |  | **0.018** |
| <= 400 | 122 (43.6%) | 93 (33.2%) |  |
| > 400 | 26 (9.3%) | 39 (13.9%) |  |
| Albumin(g/dl), n (%) |  |  | 0.588 |
| < 3.5 | 39 (13%) | 30 (10%) |  |
| >= 3.5 | 122 (40.7%) | 109 (36.3%) |  |
| Vascular invasion, n (%) |  |  | **0.002** |
| No | 123 (38.7%) | 85 (26.7%) |  |
| Yes | 45 (14.2%) | 65 (20.4%) |  |
