# Supplementary material for: D-xylose suppresses hepatocellular carcinoma progression by regulating dihydrodiol dehydrogenase and remodeling the immune microenvironment
Source: Front Immunol. 2026 Mar 13;17:1792196. doi: 10.3389/fimmu.2026.1792196 (PMC13021656; doi:10.3389/fimmu.2026.1792196)
Supplement: Supplementary file 6 [file Table2.docx]

**Table S2. Clinical information of hepatocellular carcinoma patients included in the tissue microarray cohort**

| Characteristics | Low expression of DHDH (n=43) | High expression of DHDH (n=42) | *P* value |
| --- | --- | --- | --- |
| Gender, n (%) |  |  | 0.332 |
| Male | 36 (42.4%) | 39 (45.9%) |  |
| Female | 7 (8.2%) | 3 (3.5%) |  |
| Age, mean ± sd | 48.605 ± 10.746 | 57.119 ± 8.4974 | **< 0.001** |
| Pathologic T stage, n (%) |  |  | 0.241 |
| 1 | 8 (9.4%) | 6 (7.1%) |  |
| 2 | 8 (9.4%) | 11 (12.9%) |  |
| 3 | 26 (30.6%) | 20 (23.5%) |  |
| 4 | 1 (1.2%) | 5 (5.9%) |  |
| Cirrhosis, n (%) |  |  | 0.758 |
| Yes | 28 (32.9%) | 26 (30.6%) |  |
| No | 15 (17.6%) | 16 (18.8%) |  |
| AFP(ug/L), median (IQR) | 6 (3, 133.5) | 35.5 (4.25, 2621) | **0.029** |
| Vascular invasion, n (%) |  |  | 0.505 |
| Yes | 5 (5.9%) | 7 (8.2%) |  |
| No | 38 (44.7%) | 35 (41.2%) |  |
| Ki-67 (Positive Cells, %), median (IQR) | 7.33 (4.705, 10.885) | 12.61 (8.265, 19.535) | **0.001** |
