# Supplementary material for: D-xylose suppresses hepatocellular carcinoma progression by regulating dihydrodiol dehydrogenase and remodeling the immune microenvironment
Source: Front Immunol. 2026 Mar 13;17:1792196. doi: 10.3389/fimmu.2026.1792196 (PMC13021656; doi:10.3389/fimmu.2026.1792196)
Supplement: Supplementary file 7 [file Table3.docx]

**Table S3. qPCR Primer Sequences for Target Genes**

| Gene | Species | Target sequence (5'-3') |
| --- | --- | --- |
| DHDH | Mouse | F: CCAAACGTGGAGGTGGCCTA  R: CTCGTGGTTCACACCCATCA |
| GAPDH | Mouse | F: GTCAAGGCCGAGAATGGGAA  R: CTCGTGGTTCACACCCATCA |
| DHDH | Human | F: TGCTGGACATCGGCATCTACTG  R: ATGACGCCTTCCCACGACA |
| GAPDH | Human | F: CCTCTGACTTCAACAGCGACA  R: ATGAGCTTGACAAAGTGGTCGT |
