# Supplementary material for: D-xylose suppresses hepatocellular carcinoma progression by regulating dihydrodiol dehydrogenase and remodeling the immune microenvironment
Source: Front Immunol. 2026 Mar 13;17:1792196. doi: 10.3389/fimmu.2026.1792196 (PMC13021656; doi:10.3389/fimmu.2026.1792196)
Supplement: Supplementary file 8 [file Table4.docx]

**Table S4. Prognostic analysis of samples from the TCGA cohort**

| Characteristics | Total(N) | Univariate analysis | |  | Multivariate analysis | |
| --- | --- | --- | --- | --- | --- | --- |
|  |  | Hazard ratio (95% CI) | *P* value |  | Hazard ratio (95% CI) | *P* value |
| DHDH | 373 |  |  |  |  |  |
| Low | 186 | Reference |  |  | Reference |  |
| High | 187 | 2.076 (1.455 - 2.963) | **< 0.001** |  | 1.834 (1.151 - 2.923) | **0.011** |
| Gender | 373 |  |  |  |  |  |
| Male | 252 | Reference |  |  |  |  |
| Female | 121 | 1.261 (0.885 - 1.796) | 0.200 |  |  |  |
| Age | 373 |  |  |  |  |  |
| <= 60 | 177 | Reference |  |  |  |  |
| > 60 | 196 | 1.205 (0.850 - 1.708) | 0.295 |  |  |  |
| Race | 361 |  |  |  |  |  |
| Asian | 159 | Reference |  |  |  |  |
| Black or African American | 17 | 1.585 (0.675 - 3.725) | 0.290 |  |  |  |
| White | 185 | 1.323 (0.909 - 1.928) | 0.144 |  |  |  |
| Pathologic T stage | 370 |  |  |  |  |  |
| T1 | 183 | Reference |  |  | Reference |  |
| T2 | 94 | 1.431 (0.902 - 2.268) | 0.128 |  | 1.464 (0.800 - 2.680) | 0.217 |
| T3 | 80 | 2.674 (1.761 - 4.060) | **< 0.001** |  | 2.542 (1.476 - 4.376) | **< 0.001** |
| T4 | 13 | 5.386 (2.690 - 10.784) | **< 0.001** |  | 4.547 (1.670 - 12.378) | **0.003** |
| Pathologic N stage | 258 |  |  |  |  |  |
| N0 | 254 | Reference |  |  |  |  |
| N1 | 4 | 2.029 (0.497 - 8.281) | 0.324 |  |  |  |
| Pathologic M stage | 272 |  |  |  |  |  |
| M0 | 268 | Reference |  |  | Reference |  |
| M1 | 4 | 4.077 (1.281 - 12.973) | **0.017** |  | 0.845 (0.162 - 4.397) | 0.841 |
| Tumor status | 354 |  |  |  |  |  |
| Tumor free | 202 | Reference |  |  | Reference |  |
| With tumor | 152 | 2.317 (1.590 - 3.376) | **< 0.001** |  | 1.935 (1.210 - 3.094) | **0.006** |
| AFP(ng/ml) | 279 |  |  |  |  |  |
| <= 400 | 215 | Reference |  |  |  |  |
| > 400 | 64 | 1.075 (0.658 - 1.759) | 0.772 |  |  |  |
| Albumin(g/dl) | 299 |  |  |  |  |  |
| < 3.5 | 69 | Reference |  |  |  |  |
| >= 3.5 | 230 | 0.897 (0.549 - 1.464) | 0.662 |  |  |  |
| Vascular invasion | 317 |  |  |  |  |  |
| No | 208 | Reference |  |  |  |  |
| Yes | 109 | 1.344 (0.887 - 2.035) | 0.163 |  |  |  |
